# Gambling Harm Experienced by Children Exposed to Parental Gambling: An Online Survey of Australians

**DOI:** 10.1007/s10899-023-10211-4

**Published:** 2023-05-07

**Authors:** Aino Suomi, Nina Lucas, Nicki Dowling, Paul Delfabbro

**Affiliations:** 1 Centre for Gambling Research, ANU Centre for Social Research and Methods, Australian National University, Canberra, Australia; 2 Institute of Child Protection Studies, Australian Catholic University, Canberra, Australia; 3 School of Psychology, Deakin University, Burwood, Australia; 4 School of Psychology, University of Adelaide, Adelaide, Australia

**Keywords:** Problem gambling, Gambling harm, Child development, Parenting, Child abuse & neglect, Family wellbeing

## Abstract

Although child wellbeing is known to be negatively affected by gambling, relatively little is known about the specific harms experienced by children exposed to parental gambling problems. The current study aimed to better understand gambling harm directly attributed to regular parental gambling in key areas of child wellbeing: financial, psychological, interpersonal wellbeing and intergenerational transmission of problem gambling. Using data from a national survey of Australian adults exposed to parental gambling under the age of 18 (*n* = 211), the results show that parental gambling was related significant levels of financial harm, abuse, neglect as well as relational and psychological problems as a direct result of parental gambling. The likelihood of experiencing gambling harms was positively associated with parental problem gambling severity. Harmful impacts of parental gambling as a child were also associated with a range of psychological problems in adulthood including depression, anxiety, Post-Traumatic Stress Disorder and intimate partner violence victimisation. Parental problem gambling severity was negatively associated with own lifetime gambling problems, suggesting a specific pattern of intergenerational transmission of problem gambling in children of regular, or heavy, gamblers. This research highlights the need for more supports for families with children in which at least one parent gambles regularly.

## Introduction

Gambling harms experienced by gamblers and their significant others include financial problems, physical health concerns, psychological distress, and poor family functioning (Bellringer et al., 2013; Cowlishaw & Kessler, 2016; Dowling et al., 2016a; Hodgins et al., 2007; Langham et al., 2016). Children are likely to be most vulnerable to these negative impacts, and recent data shows that over 10%, and as many as 14% of the Australian child population is exposed to parental at-risk or problem gambling (Suomi et al., 2022a; Tulloch et al., 2022). The direct impacts of parental gambling and problem gambling on children have to date only received a modest amount of attention.

A recent systematic review of 35 studies (Suomi et al., 2022b) provides evidence of six broad areas of child wellbeing that are likely to be affected by parental problem gambling. These areas are: (1) psychological (mood disorders, emotional distress); (2) relational (child-parent relationship, family dysfunction); (3) violence exposure (parental violence in the home home); (4) behavioural (conduct problems, hyperactivity); (5) financial (lack of money for food or education); and (6) untreated physical health conditions. In this review, a convergence of evidence supported the view that parental problem gambling can lead to psychological harm in children, as well as dysfunctional family relationships. In addition, 17–43% of parents with gambling problems have perpetrated child physical abuse and that children in these families are also likely to experience parental neglect (Afifi et al., 2010; Dowling et al., 2016a; Lesieur & Rothschild, 1989; Suomi et al., 2019). Similarly, previous research shows that parental problem gambling is strongly associated with poor parent–child relationships and maladaptive family environments (Suomi et al., 2022b). Only a few studies have addressed the financial impacts of gambling specifically focusing on children, that are likely to manifest as lack of food, poor housing or needing to take on financial responsibilities at a young age (Mathews & Volberg, 2013; Schluter et al., 2007, 2008). Less is known, however, about the financial and physical health impacts of parental problem gambling on children, even though these are recognised as major domains of harm in the adult gambling affected other literature (Dowling et al., 2021a; Kalischuk et al., 2006; Langham et al., 2016). In addition, the review highlighted the need for more evidence about the direct impacts of parental gambling across a broad range of child wellbeing domains (Suomi et al., 2022b).

Studies examining the impact of gambling on families also has implications for the study of the potential intergenerational transmission of gambling, whereby children of people with gambling problems develop gambling problems themselves (Dowling et al., 2016b, 2017, 2018, 2021b; Govoni et al., 1996; Gupta & Derevensky, 1997). Although children with problem gambling parents are at risk for this pattern of problem transmission, only a small proportion develop gambling problems themselves, suggesting an interplay between risk and protective factors (Dowling et al., 2016b). Known mediators of the intergenerational transmission of gambling problems include gambling expectancies, gambling motives, parental psychopathology (problem drinking and drug use), financial debts, parental separation, and child psychopathology (depression, drug use) (Dowling et al., 2016b, 2018). Moreover, family factors, such as parental involvement, single-parenthood, and a higher number of siblings have been found to buffer the intergenerational transmission of gambling problems (Dowling et al., 2017).

Empirically-derived extended list of adverse childhood events (ACE) now include parental problem gambling (Afifi et al., 2020), however, there is little evidence about the longer-term consequences of experiencing parental problem gambling as a child (Suomi et al., 2023). Other ACEs broadly known to predict a range of poor outcomes in adulthood such as physical and mental illness, drug and alcohol use, interpersonal and self-directed violence and homelessness (Bellis et al., 2019; Hughes et al., 2017; Liu et al., 2021), and it is likely that parental gambling would similarly translate to poor adult outcomes. This longer-term evidence is needed to better understand the extent of gambling harms in families and appropriately respond to it.

Taken together, it is well-established that children living with a problem gambling parent can be at risk of harm. More research is needed, however, to understand the relationship between parental gambling and the mechanisms of negative consequences for the adult child. The current study therefore aimed to examine:

1. The specific harms reported by Australian adults in childhood as a result of regular parental gambling;
2. The degree to which parent factors during childhood (parent problem gambling severity, two gambling parents, years of exposure to problem gambling, responsive parenting, and primary gambling parent gender) are associated with harms in childhood resulting from regular parental gambling;
3. The ways harms resulting from regular parental gambling in childhood relate to outcomes later in life (depression, anxiety, general health, PTSD, IPV victimisation and perpetration, alcohol abuse, smoking, drug use and lifetime gambling problems).

## Methods

### Recruitment and Sampling

The sample for the current study included 211 participants who endorsed the question: “*Has your parent(s) ever regularly participated in electronic gaming machines (pokies), race betting, sports betting, casino table games, private betting or poker for money before you were under the age of 18*?*”.* Participants were 36.1 years old, on average (SD = 13.7), and 68% of them were female, 3% were Indigenous and 9% born outside Australia. Eight percent of the participants had not completed high school, 15% had completed high school as their highest level of education, over half (54%) had a University degree and 22% had another post-school qualification. The Qualtrics online survey was open from August 2020 until February 2021 to Australian residents aged 18 years and older. Recruitment was via social media platforms and no participant remuneration was provided.

### Measures

The gambling harm, parent factors and current wellbeing outcome scales employed in this study are described in full in Table 1

**Table 1:** Gambling harm, parent factors and current wellbeing outcome scales used in the study

Construct	Measure	Description of measure
Gambling harm to children	Adaptation of the U.S. National Alcohol’s Harm to Others Survey (Kaplan et al., 2017)	8 items assessing whether the following had ever happened when participant was < 18 years of age because of their parent’s gambling (yes/no to each item): (1) You or other children in the household were physically harmed; (2) You or other children in the household were yelled at, criticized, or verbally abused; (3) You or other children were left unsupervised; (4) There was not enough money for your needs, or for those of another child (5) You or other children in the household witnessed violence; (6) Child welfare services were contacted for you or other children in the household; (7) To your knowledge, you or another child was distressed or upset about the gambling; (8) You or another child had serious problems in their relationship with this parent
Parent problem gambling severity	Adaptation of CAST-6 (Hodgins et al., 1993)	6 items assessing whether the following ever happened when participant was < 18 years of age (yes/no to each item) (1) Have you ever thought that this parent had a gambling problem (2) Did you ever encourage this parent to quit gambling? (3) Did you ever argue or fight with this parent about their gambling? (4) Have you ever heard this parent fight with others about their gambling? (5) Did you ever feel like limiting this parent's access to money for gambling? (6) Did you ever wish that this parent would stop gambling?
Responsive parenting	Parenting Style Inventory (Darling & Toyokawa, 1997), Emotional Responsiveness Subscale	5 items measuring emotional responsiveness of gambling parent during the participant’s childhood; 5-point scale (1–5)
Depression	Patient Health Questionnaire (PHQ-2; Kroenke, Spitzer, & Williams, 2003)	2-items indicating depression symptoms in the past two weeks; 4-point scale (0–3). Responses were summed; higher scores indicate more depression symptoms
Anxiety	Generalized Anxiety Disorder scale (GAD-2; Kroenke et al., 2007)	2-items indicating anxiety symptoms in past two weeks; 4-point scale (0–3). Responses were summed; higher scores indicate more anxiety symptoms
General health	SF-36 (Ware & Sherbourne, 1992)	Single items indicating current general health; 5-item scale (1–5). Higher scores indicated better health
Post-traumatic stress disorder	Primary Care PTSD Screen (PC-PTSD; Cameron & Gusman, 2003)	4-items indicating PTSD symptoms in the last month; yes/no to each item. Final scores were a count of endorsements
IPV victimisation	Short-Form Revised Conflict Tactics Scale, Physical Assault subscale (Straus et al., 1996)	3-items indicating acts of physical violence by a partner in the past year; yes/no to each item. Any item endorsement indicated intimate partner victimisation
IPV perpetration	Short-Form Revised Conflict Tactics Scale, Physical Assault subscale (Straus et al., 1996)	3-items of indicating acts of physical violence against a partner in the past year; yes/no to each item. Any item endorsement indicated intimate partner perpetration
Alcohol Abuse	Alcohol use Disorders Identification Test (AUDIT-C)	Single item indicating frequency of having six or more drinks on one occasion; 5-point scale from (0–4). Scores of 1 + indicated alcohol abuse
Smoking	Smoking item from the ASSIST (WHO Assist Working Group, 2002)	Single item indicating use of tobacco products in past 3 months; yes/no response
Drug use	Single-Question Screening Test for Drug Use (Smith et al., 2010)	Single item indicating frequency of using an illegal drug or a prescription drug for a non-medical reason in the past year. Responses dichotomised into any/no drug use
Lifetime gambling problems	NODS-CLiP (Volberg, 2011)	3-items indicating lifetime symptoms of problem gambling; yes/no to each item. Final scores were a count of endorsements

***Gambling harm.*** Gambling harm to children was assessed using six items adapted from the U.S. National Alcohol’s Harm to Others Survey (Kaplan et al., 2017), with two additional items (7 and 8 below) identified in the literature as key types of gambling harm experienced by children due to parental gambling. This measure has not been previously validated in the gambling context and the adaptation was developed for the purposes of this study. The questionnaire used the following wording: “*Thinking about when you were a child, did the following ever happen to you because of your parent's gambling*?” with yes/no response options. The eight harms on this adapted scale, herein called the Gambling Harm to Children Scale, assessed were: (1) physical abuse; (2) verbal abuse; (3) being left unsupervised; (4) not enough money; (5) witnessing violence; (6) child welfare services contacted; (7) distress or upset; and (8) relationship problems with the gambling parent.

***Parent factors.*** Parent problem gambling severity was assessed using an adaptation of the Children of Alcoholics Screening Test (CAST-6; Hodgins et al., 1993). Wording of items was adapted to parental gambling and endorsements were counted to create a continuous symptom severity score (Hodgins et al., 1993). Respondents also indicated whether they had two gambling parents (yes/no). Participants also reported the years exposed to parental gambling under the age of 18 years. Responsive parenting of the gambling parent was assessed via the Emotional Responsiveness subscale from the Parenting Style Inventory (Darling & Toyokawa, 1997). Participants with two gambling parents were also required to indicate the primary gambling parent gender (male, female) (i.e., the parent who they thought engaged in more severe gambling).

***Current wellbeing outcomes.*** Participant depression was assessed via the Patient Health Questionnaire (PHQ-2; Kroenke et al., 2003); anxiety via the Generalized Anxiety Disorder scale (GAD-2; Kroenke et al., 2007); general health via the first item on SF-36 (Ware & Sherbourne, 1992); Post-Traumatic Stress Disorder (PTSD) via the Primary Care PTSD Screen (PC-PTSD; Cameron & Gusman, 2003) and intimate partner violence (IPV; victimisation and perpetration) via the Physical Assault subscale of the Short Form Revised Conflict Tactics Scale (CTS2; Straus et al., 1996). For other addictive behaviours, participant alcohol abuse was assessed via a short form of the Alcohol use Disorders Identification Test (AUDIT-C; Bush et al., 1998); smoking via an item from the Alcohol, Smoking and Substance Use Involvement Screening Test (ASSIST; WHO Assist Working Group, 2002); and drug use via the Single-Question Screening Test for Drug Use (Smith et al., 2010). Participants’ own lifetime gambling problems was assessed using the NODS-CLiP (Volberg et al., 2011).

### Analysis

Data analyses were conducted using STATA version 14.2. Descriptive statistics were employed to identify participants’ demographic profiles and the specific gambling harms they experienced during childhood. Spearman correlations examined bivariate associations between gambling harm items and parent factors. Significantly correlated variables (with any harm item) were then entered into a series of multivariable logistic regressions to examine the independent predictors of each harm item after adjusting for covariates. Spearman correlations also examined associations between each harm item, gambling parent factors and participants’ current (adult) wellbeing outcomes. Significantly correlated variables (with any outcome measure) were then entered into a series of multivariable models (negative binomial and logistic regressions) to predict scores on each current wellbeing outcome measure after adjusting for covariates. Covariates included in the models were participant age, gender, Indigenous status, whether they were born in Australia, and highest level of education.^1^ Missing data, which ranged from 0.5 and 24.1 percent across the variables, were identified as Missing at Random (MAR). Pairwise deletion of missing data was employed for the bivariate analyses and complete case analysis was employed for the multivariate models. Violations of normality were addressed with log transformations, however the analytical approach used in the current study is robust for non-normal data (Spearman’s Rho, negative binomial and logistic models) (Elhai et al., 2008).

## Results

### Gambling Harm to Children

Table 2 describes the descriptive statistics for the study outcome variables with total n’s, and shows that the most commonly reported harms attributed to regular parental gambling in childhood (on the Gambling Harm to Children Scale) were emotional distress, difficulties in the relationship with the gambling parent, not having enough money, and being left unsupervised. Verbal abuse due to gambling was also common, reported by nearly half of participants, and witnessing violence was reported by around one-third of participants. The more severe types of harm, physical abuse and child welfare services being called due to parental gambling, were the least commonly reported harms.

**Table 2:** Description of the variables used in the analysis

				Total *n*
*Gambling harm to children*				
1		Physical abuse, *n* (%)	31 (17.1)	181
2		Verbal abuse, *n* (%)	89 (48.1)	184
3		Left unsupervised, *n* (%)	92 (50.0)	185
4		Not enough money, *n* (%)	93 (51.4)	181
5		Witness violence, *n* (%)	63 (34.8)	181
6		Child welfare, *n* (%)	12 (6.6)	183
7		Emotional distress, *n* (%)	108 (58.1)	186
8		Relationship problems, *n* (%)	97 (52.5)	186
*Parent factors*				
Parent problem gambling severity	M (SD)		3.1 (2.4)	189
	Score 0–2 (non-problem gambling), n (%)		78 (41.3)	
	Score 3–6 (problem gambling), n (%)		111 (58.7)	
Two gambling parents, *n* (%)			49 (25.7)	191
Years exposed to parental gambling, *M* (SD)			12.2 (6.0)	190
Responsive parenting, *M* (SD)^a^			3.0 (1.05)	190
Primary gambling parent gender (male), *n* (%)			130 (64.4)	202
*Participant current wellbeing outcomes*				
Depression, M (SD)			2.0 (2.0)	163
Anxiety, M (SD)			2.3 (2.1)	165
General health (good/fair/poor), *n* (%)			117 (70.9)	165
Post Traumatic Stress Disorder, M (SD)			1.7 (1.5)	165
IPV Victimisation, *n* (%)			62 (28.3)	162
IPV Perpetration, *n* (%)			41 (25.4)	162
Alcohol abuse, *n* (%)			72 (43.6)	165
Smoking, *n* (%)			45 (27.6)	164
Drug use, *n* (%)			24 (14.6)	163
Lifetime gambling problems, M (SD)			0.87 (1.2)	211

Proportion of missing data varies across the variables, as indicated by the Total *n*’s in the last column

### Predictors of Gambling Harm to Children

Table 3 shows the bivariate associations between the Gambling Harm to Children Scale items and parent factors: parent problem gambling severity, two gambling parents (as opposed to one), years exposed to parental gambling, responsive parenting, and primary gambling parent gender. Parental problem gambling severity was significantly positively associated with each of the harm items, while responsive parenting was significantly negatively associated with each of the harm items, with the exception of child welfare services being contacted. Two gambling parents was positively associated with being left unsupervised, while female parents as the primary gambler was associated with a higher likelihood of being left unsupervised, emotional distress, and relationship problems. The number of years exposed to parental gambling was not associated with any harm item.

**Table 3:** Bivariate correlations between gambling harm items and parent factors

	1	2	3	4	5	6	7	8	9	10	11	12
1 Physical abuse	1.00											
2 Verbal abuse	.49*	1.00										
3 Left unsupervised	.21*	.38*	1.00									
4 Not enough money	.37*	.50*	.28*	1.00								
5 Witness violence	.65*	.60*	.25*	.35*	1.00							
6 Child welfare	.12	.24*	.23*	.22*	.28*	1.00						
7 Emotional distress	.34*	.51*	.37*	.53*	.32*	.14	1.00					
8 Relationship problems	.41*	.55*	.42*	.50*	.49*	.22*	.61*	1.00				
9 Parent problem gambling severity	.28*	.49*	.45*	.54*	.30*	.19*	.77*	.62*	1.00			
10 Two gambling parents	.11	.04	.22*	.03	.08	.00	.03	.05	.01	1.00		
11 Years exposed to parental gambling	.12	.02	.02	.08	− .04	− .36	.13	.05	.06	− .06	1.00	
12 Responsive parenting	− .33*	− .44*	− .35*	− .33*	− .36*	.03	− .36*	− .50*	− .30*	1.00	− .02	1.00
13 Primary gambling parent gender^a^	− .02	− .14	− .27*	− .11	− .02	− .04	− .24*	− .17*	− .32*	.13	.14	0.02

* = *p* < .05; ^a^ = negative association indicates higher likelihood for female parents

Significantly correlated variables were entered into a series of logistic regression models predicting each type of harm, with participant age, gender, Indigenous status, and country of birth employed as covariates. The multivariable models in Table 4 showed that with the exception of child welfare calls, parental problem gambling severity significantly positively predicted each type of harm after controlling for covariates. Participants with two gambling parents were more likely to have been left unsupervised due to parental gambling compared to those with only one gambling parent. Responsive parenting also consistently negatively predicted each of the gambling harm items, with the exception of child welfare calls. The gender of the primary gambling parent was not associated with any of the harm items.

**Table 4:** Multivariable models predicting gambling harm to children items: Coefficients and [95% CIs] (**Bolded** Cells Indicate *p* < .05)

Predictor variables	Physical abuse	Verbal abuse	Left unsupervised	Not enough money	Witness violence	Child welfare	Emotional distress	Relation-ship problems
Parent problem gambling severity	**0.41 [0.11, 0.72]**	**0.48 [0.27, 0.69]**	**0.32 [0.13, 0.51]**	**0.63 [0.39, 0.86]**	**0.43 [0.20, 0.66]**	0.34 [-0.17, 0.85]	**1.06 [0.72, 1.39]**	**0.66 [0.42, 0.91]**
Two gambling parents (ref one)	0.50 [− 0.66, 1.66]	− 0.34 [− 1.33, 0.64]	**1.32 [0.31, 2.33]**	− 0.08 [− 1.10, 0.94]	− 0.04 [− 1.00, 0.93]	0.21 [− 2.34, 2.29]	− 0.14 [− 1.52, 1.24]	0.09 [− 1.05, 1.22]
Responsive parenting	**− 1.00 [− 1.65, − 0.35]**	**− 0.80 [− 1.27, 0.34]**	**− 0.80 [− 1.25, 0.35]**	**− 0.56 [− 1.02, − 0.10]**	**− 0.62 [− 1.08, − 0.17]**	0.17 [− 0.70, 1.00]	**− 0,58 [− 1.18, 0.03]**	**− 1.31 [− 1.91, − 0.71]**
Primary gambling parent gender (ref female)	0.56 [− 0.54. 1.66]	− 0.30 [− 1.16, 0.56]	− 0.27 [− 1.12, 0.58]	0.50 [− 0.40, 1.41]	0.73 [− 0.17, 1.62]	1.00 [− 1.00, 3.00]	− 0.34 [− 1.53, 0.85]	1.52 [− 0.19, 1.28]
Prob > chi2	0.0003	0.0000	0.0000	0.0000	0.0000	0.2608	0.0000	0.0000
Pseudo R2	0.2281	0.2996	0.2626	0.3268	0.2029	0.1536	0.5612	0.4347

Models are adjusted for participant age, gender, Indigenous status and country of birth

### Predictors of Current (Adult) Wellbeing

Table 5 shows bivariate associations between the Gambling Harm to Children items, parent factors, and participant current wellbeing outcomes. Gambling harm items were associated with all current wellbeing outcomes, with the exception of general health and smoking. Parent problem gambling was positively associated with current PTSD and negatively associated with participant own lifetime gambling problems. Having two gambling parents was positively related to IPV victimisation and responsive parenting was negatively related with current depression, anxiety and PTSD and both IPV victimisation and perpetration. Having a female primary gambling parent was related to higher scores of current depression, anxiety, and IPV victimisation and perpetration.

**Table 5:** Bivariate Correlations Between Gambling Harm Items, Parent Factors and Current Wellbeing Outcomes

	Dep-ression	Anxiety	General health	PTSD	Alcohol abuse	Smoking	Drug use	IPV Victim-isation	IPV perp-etration	Lifetime gambling problems
*Gambling harm to children-items*										
Physical abuse	.17*	.23*	.13	.33*	.01	.02	− .04	.12	.17*	− .00
Verbal abuse	.20*	.28*	− .09	.39*	− .07	.06	.02	.24*	.19*	− .10
Unsupervised	.07	.28*	.05	.24*	.13	.12	.03	.22*	.23*	− .02
No money	.02	.16	− .04	.20*	.09	.03	− .19*	.09	.06	− .11
Witness violence	.16*	.22*	− .08	.26*	− .01	.04	.00	.08	.15	− .01
Child welfare call	.16*	.16*	− .10	.22*	.17*	.10	.05	.09	.18*	− .02 −
Emotional distress	.04	.10	− .04	.24*	.01	− .01	− .09	.21*	.16*	− .22*
Relationship problems	.07	.23*	− .03	.28*	− 0.09	− .01	− .05	.28*	.14	− .22*
*Gambling parent factors*										
Parent PG severity	.02	.14	.07	.24*	.03	− .06	− .12	.11	.06	− .26*
Two gambling parents	.03	− .09	− .11	.02	.09	.04	− .05	.24*	.09	.10
Years exposed to parental gambling	− 0.05	− 0.05	− 0.05	0.01	0.07	− 0.02	0.19	0.19	0.01	0.01
Responsive parenting	− .20*	− .20*	.09	− .27*	.11	− .06	− .04	− .29*	− .17*	.08
Primary gambling parent gender^a^	− .11*	− .19*	.14	− .11	.09	.03	.07	− .16*	− .20*	− .06

*p < .05; *PG*  problem gambling; *PTSD*  post traumatic stress disorder, *IPV*  intimate partner violence

The harm items and gambling parent factors that were significantly correlated with current wellbeing outcomes were then examined in a series of multivariate models: depression, anxiety, PTSD, IPV victimisation, IPV perpetration, alcohol abuse, drug use, and lifetime gambling problems (Table 6). These models indicate that, after adjusting for the covariates, child welfare calls due to parental gambling and low levels of responsive parenting significantly predicted current depression symptoms. Having a gambling mother (as opposed to having a gambling father) positively predicted current anxiety symptoms, IPV perpetration, and lifetime gambling problems. Verbal abuse due to parental gambling positively predicted current PTSD symptoms. Verbal abuse, maternal gambling, and low levels of responsive parenting predicted IPV victimisation. There were no significant predictors of current alcohol abuse or drug use in the multivariable models.

**Table 6:** Multivariable Models Predicting Current Wellbeing Outcomes (**Bolded** Cells Indicate *p* < .05)

Predictor variables	Depression [95%CI]	Anxiety [95%CI]	PTSD [95%CI]
Physical abuse	0.43 [− 0.36, 1.22]	0.23 [− 0.32, 0.78]	0.39 [− 0.17, 0.94]
Verbal abuse	0.40 [− 0.24, 1.05]	0.27 [− 0.18, 0.73]	**0.55 [0.07, 1.03]**
Left unsupervised	− 0.24 [− 0.73, 0.25]	0.09 [− 0.28, 0.45]	0.15 [− 0.24, 0.54]
Not enough money	− 0.29 [− 0.92, 0.33]	0.03 [− 0.41, 0.47]	0.03 [− 0.44, 0.50]
Witnessed violence	− 0.18 [− 0.91, 0.56]	0.01 [− 0.52, 0.54]	− 0.15 [− 0.72, 0.41]
Child welfare	**1.20 [0.14, 2.27]**	0.30 [− 0.39, 0.98]	0.53 [− 0.14, 1.21]
Emotional distress	− 0.37 [− 1.13, 0.38]	− 0.30 [− 0.82, 0.21]	− 0.12 [− 0.64, 0.41]
Relationship problems	− 0.18 [− 0.84, 0.47]	0.19 [− 0.31, 0.67]	− 0.26 [− 0.78, 0.25]
Parent problem gambling severity	0.22 [− 0.15, 0.20]	− 0.10 [− 0.13, 0.10]	0.02 [− 0.13, 0.04]
Two gambling parents	0.03 [− 0.44, 0.51]	− 0.34 [− 0.72, 0.04]	0.02 [− 0.35, 0.40]
Responsive parenting	** − 0.27 [− 0.51, − 0.02]**	− 0.07 [− 0.25, 0.11]	− 0.14 [− 0.33, 0.04]
Primary gambling parent gender (ref female)	− 0.36 [− 0.83, 0.10]	** − 0.37 [− 0.72, − 0.04]**	− 0.14 [− 0.49, 0.20]
Model fit	Pseudo *R*^2^ = 0.03 Prob > chi^2^ = 0.52	Pseudo *R*^2^ = 0.06 Prob > chi^2^ = 0.03	Pseudo *R*^2^ = 0.06 Prob > chi^2^ = 0.06

Predictor variables	IPV victimisation	IPV perpetration	Alcohol abuse	Drug use	Lifetime gambling problems
Physical abuse	0.06 [− 1.51, 1.62]	0.55 [− 1.13, 2.23]	0.16 [− 1.38, 1.70]	0.04 [− 2.0, 2.0]	0.33 [− 1.01, 1.77]
Verbal abuse	**1.39 [0.09, 2.68]**	0.48 [− 0.92, 1.88]	− 0.89 [− 2.01, 0.28]	1.43 [− 0.26, 3.12]	− 0.05 [− 1.16, 1.07]
Left unsupervised	0.09 [− 0.96, 1.14]	0.67 [− 0.44, 1.78]	0.64 [− 0.32, 1.59]	0.12 [− 0.19, 1.42]	0.34 [− 0.47, 1.15]
Not enough money	− 0.72 [− 2.01, 0.56]	− 0.43 [− 1.73, 0.86]	0.33 [− 0.79, 1.44]	− 1.80 [− 3.56, 0.04]	− 0.38 [− 1.51, 0.74]
Witnessed violence	− 0.83 [− 2.38, 0.72]	− 0.48 [− 2.15, 1.19]	− 0.01 [− 1.39, 1.38]	− 0.01 [− 1.89, 1.88]	0.72 [− 0.62, 2.07]
Child welfare	1.64 [− 0.42, 3.72]	1.41 [− 0.75, 3.57]	1.77 [− 0.62, 4.16]	− 1.09 [− 1.71, 3.88]	− 0.48 [− 2.40, 1.44]
Emotional distress	0.50 [− 0.94, 1.94]	1.49 [− 0.23, 3.21]	0.36 [− 0.93, 1.66]	1.15 [− 1.62, 1.92]	− 0.20 [− 1.31, 0.91]
Relationship problems	0.08 [− 1.28, 1.45]	− 0.54 [− 2.05, 0.96]	− 0.58 [− 1.86, 0.71]	− 0.65 [− 2.78, 1.47]	− 0.58 [− 1.78, 0.62]
Parent problem gambling severity	− 0.02 [− 1.28, 1.45]	0.26 [-0.60, 0.09]	0.10 [− 0.19, 0.39]	− 0.26 [− 0.73, 0.21]	− 0.11 [− 0.48, 0.38]
Two gambling parents	**1.21 [0.20, 2.22]**	− 0.17 [− 1.22, 0.88]	0.48 [− 0.44, 1.40]	0.08 [− 1.21, 1.36]	− 0.85 [− 1.65, 0.05]
Responsive parenting	** − 0.64 [− 1.17, − 0.11]**	− 0.17 [− 0.70, 0.37]	− 0.02 [− 0.48, 0.44]	− 0.77 [− 1.47, 0.08]	− 0.06 [− 0.48, 0.35]
Primary gambling parent gender (ref female)	− 0.24 [− 1.17, 0.69]	** − 1.11 [− 2.10, − 0.12]**	0.41 [− 0.48, 1.29]	0.38 [− 0.82, 1.57]	** − 0.85 [− 1.65, − 0.05]**
Model fit	Pseudo *R*^2^ = 0.21 Prob > chi^2^ = 0.00	Pseudo *R*^2^ = 0.14 Prob > chi^2^ = 0.26	Pseudo *R*^2^ = 0.11 Prob > chi^2^ = 0.30	Pseudo *R*^2^ = 0.16 Prob > chi^2^ = 0.30	Pseudo *R*^2^ = 0.10 Prob > chi^2^ = .002

Models are adjusted for participant age, gender, Indigenous status, country of birth, and education

## Discussion

Building on recent literature on parental problem gambling and child wellbeing (Suomi et al., 2022a, b, 2023; Tulloch et al., 2022), the current study is the first to concurrently link a broad range of specific harms experienced by children directly to regular parental gambling: (1) physical abuse; (2) verbal abuse; (3) being left unsupervised; (4) not having enough money; (5) witnessing violence, (6) child welfare call; (7) distress or upset; (8) problems in parent–child relationship. Of these, participants most commonly endorsed relational and emotional harms, followed by not having enough money and being left unsupervised.

The current findings specifically highlight that parental problem gambling severity was positively related to all types of gambling harm experienced by the participants when they were children, apart from child welfare calls, after adjusting for other parent factors and covariates. These results are not surprising given the interrelated nature of gambling harm and problem gambling severity (Cowlishaw et al., 2019; Delfabbro & King, 2019). The presence of a second gambling parent increased the likelihood of being left unsupervised, which is also consistent with reports from qualitative studies on the physical and emotional absence of problem gambling parents (Darbyshire, 2001). Furthermore, responsive parenting was negatively associated with gambling harm experienced by children, after adjusting for other parent factors and covariates. Previous evidence suggests that more positive parenting styles can potentially mitigate the degree of intergenerational transmission of problem gambling and (Dowling et al., 2017). It is possible that more responsive parenting styles also protect children from the harmful effects of family conflict or parental absence related to problem gambling, but these findings need further examination using larger samples and longitudinal design.

The current study builds on previous research showing that exposure to parental problem gambling is related to higher rates of mental health problems in children (Goodwin et al., 2017; Martyres & Townshend, 2016; Salonen et al., 2016; Vitaro et al., 2008). Our findings significantly add on this literature by showing that the longer term mental health consequences of parental gambling are more strongly associated with the types of gambling harm, rather than parental problem gambling severity. Analysis of these associations yielded several patterns: after controlling for parental problem gambling, exposure to child welfare services due to parental gambling as a child increased the likelihood of current depression symptoms, and verbal abuse due to parental gambling increased the likelihood of current PTSD symptoms. PTSD has been previously linked to problem gambling (Biddle et al., 2005; Nower et al., 2015) but not to exposure to parental problem gambling. The high likelihood of childhood trauma due to general instability in families in which a parent has a gambling problem may contribute to this association (Turner et al., 2012).

Consistent with previous research on intergenerational transmission of problem gambling, maternal gambling in the current study was associated with a higher likelihood of participants’ own lifetime gambling problems (Dowling et al., 2017). The current study extends these findings also showing that maternal gambling was associated with current anxiety and increased likelihood of being a victim of intimate partner violence as an adult, however, the mechanism by which maternal factors are more salient compared to paternal factors needs more exploring. Similar findings have been found in other contexts, whereby maternal psychopathology, but not paternal, negatively impacts on child health and wellbeing outcomes (Harold et al., 2012). While problem gambling is associated with both IPV victimisation and perpetration and child abuse (Dowling et al., 2016c; Roberts et al., 2018; Suomi et al., 2013, 2019), our findings also report factors associated with current IPV victimisation in adult children of problem gamblers, demonstrating specific intergenerational patterns associated with problem gambling. Responsive parenting was negatively associated with experience of gambling harms as a child, and it also decreased the likelihood of adult depression and IPV victimisation in children of regular gamblers. Positive parenting has been previously linked as a protective factor from the intergenerational transmission of problem gambling, but not from other negative consequences of parental gambling (Dowling et al., 2017).

## Limitations

There are a number of methodological limitations in this research that should be taken into account when interpreting the findings. First, given the absence of validated measures to assess affected other’s gambling severity, the current study is the first to adapt validated tools to measuring gambling harm to children caused by parental gambling: the Alcohol’s Harm to Children scale (Kaplan et al., 2017) and the Children of Alcoholics screening Test (CAST-6; Hodgins et al., 1993). Given the significant overlap on the harms to others attributed to problem gambling and problematic alcohol use, adaptations of measurement tools from alcohol research are widely used in this context (e.g., Orford et al., 2005, 2010). The current study is the first to address wide range of specific harms experienced by children as a direct result of their parent’s gambling, however, future research should validate and test such tools using comprehensive psychometric testing to further advance the research in this important area. Both of these measures were initially developed for family members of alcoholics to measure the severity of parental alcohol abuse (CAST-6) and its impacts on children (Alcohol’s Harm to Children scale). These measures rely on information about inferred problem gambling in parents, thus there is a potential for under-reporting in the current sample. Second, the study used a self-selected convenience sample, thus the findings are not representative of the broader Australian general population. Children of regular gamblers were recruited to maximise the occurrence of harm, and over half of the sample scored above 3 on the adapted CAST-6 (Hodgins et al., 1993) that is used as a cut-off to indicate problematic alcohol use. Given the CAST-6 is not validated for gambling context, these findings must be interpreted with caution. Third, the analysis of parental gambling and harm to children used retrospective self-report data, which is a well-established methodological approach in research on adverse childhood experiences (see for example Australian Child Maltreatment Study; Mathews et al., 2021). This, however, can translate to over- or underreporting on some of the key outcome measures: participants may have been limited in their ability to isolate harms caused by parental gambling from those with other contributing factors (such as mental health difficulties), or may have been unaware of their parents gambling if it occurred when they were very young. Despite these limitations, the current study provides new novel information about the patterns of harm experienced by children as a direct consequence of parental gambling.

## Clinical and Service Implications

Given the high rates of psychosocial problems in children exposed to parental problem gambling, there is a need for more consistent approaches to the assessment and treatment of children in these families. Current evidence specifically warrants more systematic identification of emotional and relational problems in children of people with gambling problems, and provision of early and targeted interventions (Suomi et al., 2022a). As some evidence-based programs targeting the socio-emotional wellbeing of adult children of gamblers already exist (Kourgiontakis et al., 2016), making these supports more readily available for parents who present to gambling treatment is needed.

Concerns about child wellbeing are a major help-seeking trigger for people with gambling problems and their spouses (Rodda et al., 2017, 2019). Thus, raising public awareness about gambling harm on children may encourage more parents into treatment. While family interventions focusing on coping skills of affected others show promising evidence (Hodgins et al., 2007; Orford et al., 2010; Rychtarik & McGillicuddy, 2006), there is a lack of interventions specifically targeting family members’ wellbeing, including children (Dowling, 2020; Dowling et al., 2021b; Kourgiantakis et al., 2021; Rodda et al., 2019).

The current results encourage the development of better service coordination to address the harm from parental problem gambling on children. Examples of these exist in alcohol, drug and mental health inter-service collaboration that ensure the wellbeing of children affected by parental addictions and mental health issues are appropriately preserved (Dowling et al., 2010). As such, problem gambling services may benefit from referral protocols to child-specific services or building capacity to provide such services themselves. A high level of integration of services encompassing assessment, referral, intervention, and post-intervention support can be used to promote better outcomes for children living in problem gambling families.

## Research Implications

The current study highlights a need for further work examining the harms experienced by children and how the harms might be related to general family dynamics. Building on the methodology used in the current study, future studies should develop and validate measures to reliably assess parental gambling severity as well as harms experienced by children. Large-scale quantitative and in-depth qualitative data, using multiple informants and a wide range of gambling impacts is needed to understand the specific mechanism between parental gambling and harm experienced by children. An important way in which future studies might build upon this work is to examine the concordance between different respondent groups including parents and child-parent dyads. Most importantly, interviewing children of people with gambling problems while, or immediately after, they are exposed to parental gambling may be useful to comprehensively gain insights into the experiences of children in problem gambling families. However, ethical aspects of collecting data from vulnerable children who may be experiencing a crisis or who may have been exposed to trauma need consideration.

## Conclusions

The current study presents one of the few empirical studies focusing on the broad ranging impacts of regular parental gambling experienced by children. The results of the study highlight the intertwined nature of adverse childhood experiences and regular parental gambling, particularly related to child neglect, abuse and various types of trauma and their long-lasting consequences. The current study provides data on multiple and specific areas of child wellbeing, from the perspectives of individuals exposed to regular parental gambling as children. It shows the complex nature of dynamics related to gambling in families but also points to multiple opportunities for supports and intervention that may improve the wellbeing of families and children exposed to gambling harm. The results of the project can be used to inform multi-sectoral service approaches that are currently needed to adequately address the negative impacts of parental gambling in families with children.

## Data Availability

The data will be made available on reasonable request.

## Footnotes

1 Education was excluded from the first set of multivariate models that only asked about childhood experiences that would have occurred prior to completing formal education.
